# A personalized biomedical risk assessment infographic for people who smoke with COPD: a qualitative study

**DOI:** 10.1186/s13722-021-00283-1

**Published:** 2022-01-06

**Authors:** Samir Gupta, Puru Panchal, Mohsen Sadatsafavi, Parisa Ghanouni, Don Sin, Smita Pakhale, Teresa To, Zafar Zafari, Laura Nimmon, for the Canadian Respiratory Research Network

**Affiliations:** 1 Keenan Research Center, Li Ka Shing Knowledge Institute, St. Michael’s Hospital, University of Toronto, Toronto, ON Canada; 2 Division of Respirology, Department of Medicine, St. Michael’s Hospital, Suite 6044, Bond Wing, 30 Bond St, Toronto, ON M5B 1W8 Canada; 3 Michael G. DeGroote School of Medicine, McMaster University, Hamilton, ON Canada; 4 Respiratory Evaluation Sciences Program, Collaboration for Outcomes Research and Evaluation, Faculty of Pharmaceutical Sciences, University of British Columbia, Vancouver, BC Canada; 5 UBC Centre for Heart Lung Innovation, St Paul’s Hospital, Providence Building, Vancouver, BC Canada; 6 Division of Respiratory Medicine, Faculty of Medicine, The University of British Columbia, Vancouver, BC Canada; 7 Faculty of Health, School of Occupational Therapy, Halifax, NS Canada; 8 Division of Respiratory Medicine, The Ottawa Hospital, Ottawa, ON Canada; 9 Child Health Evaluative Sciences, Research Institute, The Hospital for Sick Children, Toronto, ON Canada; 10 Department of Pharmaceutical Health Services Research, University of Maryland School of Pharmacy, Baltimore, MD USA; 11 Department of Occupational Science and Occupational Therapy, University of British Columbia, Vancouver, BC Canada; 12 Centre for Health Education Scholarship, University of British Columbia, Vancouver, BC Canada

**Keywords:** Behaviour change, Biomedical risk assessment, Smoking cessation, COPD, Infographic, Motivation to quit, Qualitative content analysis

## Abstract

**Background:**

Chronic obstructive pulmonary disease (COPD) causes 3 million deaths each year, yet 38% of COPD patients continue to smoke. Despite proof of effectiveness and universal guideline recommendations, smoking cessation interventions are underused in practice. We sought to develop an infographic featuring personalized biomedical risk assessment through future lung function decline prediction (with vs without ongoing smoking) to both prompt and enhance clinician delivery of smoking cessation advice and pharmacotherapy, and augment patient motivation to quit.

**Methods:**

We recruited patients with COPD and pulmonologists from a quaternary care center in Toronto, Canada. Infographic prototype content and design was based on best evidence. After face validation, the prototype was optimized through rapid-cycle design. Each cycle consisted of: (1) infographic testing in a moderated focus group and a clinician interview (recorded/transcribed) (with questionnaire completion); (2) review of transcripts for emergent/critical findings; and (3) infographic modifications to address findings (until no new critical findings emerged). We performed iterative transcript analysis after each cycle and a summative qualitative transcript analysis with quantitative (descriptive) questionnaire analysis.

**Results:**

Stopping criteria were met after 4 cycles, involving 20 patients (58% male) and 4 pulmonologists (50% male). The following qualitative themes emerged: Tool content (infographic content preferences); Tool Design (infographic design preferences); Advantages of Infographic Messaging (benefits of an infographic over other approaches); Impact of Tool on Determinants of Smoking Cessation Advice Delivery (impact on barriers and enablers to delivery of smoking cessation advice in practice); and Barriers and Enablers to Quitting (impact on barriers and enablers to quitting). Patient Likert scale ratings of infographic content and format/usability were highly positive, with improvements in scores for 20/21 questions through the design process. Providers scored the infographic at 77.8% (“superior”) on the Suitability Assessment of Materials questionnaire.

**Conclusions:**

We developed a user preference-based personalized biomedical risk assessment infographic to drive smoking cessation in patients with COPD. Our findings suggest that this tool could impact behavioural determinants of provider smoking-cessation advice delivery, while increasing patient quit motivation. Impacts of the tool on provider care, patient motivation to quit, and smoking cessation success should now be evaluated in real-world settings.

**Supplementary Information:**

The online version contains supplementary material available at 10.1186/s13722-021-00283-1.

## Background

Chronic obstructive pulmonary disease (COPD) affects over 300 million people and accounts for over 3 million deaths globally each year [1, 2]. Despite that smoking cessation has the greatest capacity to alter the natural history of COPD [3], 38% of patients with COPD continue to smoke daily [4].

Previous studies have demonstrated that brief smoking cessation advice offered by physicians significantly improves quit rates, with more intensive interventions, particularly those featuring the use of smoking cessation pharmacotherapies proving additionally effective [5, 6]. Although international guidelines consistently recommend smoking cessation counseling by healthcare professionals [7], even brief smoking cessation interventions are underused in actual practice. Only 37% of US [8] and 54% of Canadian people who smoke [9] who had a visit with a physician during the prior year had been advised to quit smoking.

Although it has long been known that, on average, lung function drops more rapidly in people who smoke than in those who do not [10], we recently developed a *personalized* risk calculator for forced expiratory volume in one second (FEV1) decline in COPD (http://resp.core.ubc.ca/ipress/FEV1Pred) [11]. Given that smoking status is the only modifiable risk factor affecting an individual’s future rate of FEV1 decline [11], this created an opportunity to calculate and report the differing personalized impact of ongoing smoking versus smoking cessation on future FEV1 in people with COPD who smoke. Such assessments—called personalized biomedical risk assessments—have previously been shown to effectively improve health behaviours. For instance, personalized estimates of future melanoma risk have been shown to increase use of sun protection [12], a personal skin photography intervention improved skin examination behaviour [13], and individualized genetic risk estimates enabled improvements in diet [14].

Although effects on smoking cessation have been mixed [15], providing people who smoke with pictures of their own atherosclerotic plaques improved cessation rates by threefold [16], and providing people who smoke with COPD with a personalized spirometric “lung age” improved short- and long-term quit rates [17, 18]. It is also established that infographics combining visual and text information may improve patients’ grasp of health information and enhance decision-making capacities, thereby aiding in modifying patients’ health behaviours [19].

Applying the knowledge-to-action framework for health behaviour change [20], we hypothesized that an infographic featuring a personalized biomedical risk assessment in the form of future FEV1 prediction could address known barriers and leverage enablers to clinician delivery of smoking cessation advice and prescription of smoking cessation pharmacotherapy, while also augmenting patient motivation to quit [21].

Herein, we report on patient and clinician stakeholder preferences for such a personalized lung function decline prediction infographic, and the systematic development process employed to optimize its design and content in accordance with stakeholder preferences.

## Methods

We developed the infographic using a rapid-cycle design process, which involved identifying and addressing user preferences and practical problems with the tool via incremental analysis [22, 23]. Preferences surrounding format, content and overall design of the infographic were assessed through serial semi-structured focus groups with patients, and serial interviews with pulmonologists.

### Study setting and population

The study took place at Unity Health (St. Michael’s Hospital)—a quaternary care center in Toronto, Canada. We recruited patients with physician-diagnosed COPD from an existing respiratory disease database, by telephone. Inclusion criteria were as follows: age ≥ 35 years, smoking history of ≥ 10 years, post-bronchodilator forced expiratory volume in one second/forced vital capacity ratio (FEV1/FVC) < 0.7, FEV1 ≥ 30% predicted, and ability to read, write, and speak English. We employed purposive sampling to achieve sample heterogeneity with respect to sex, disease duration, and socio-economic background. Given that some current smokers are not motivated to quit and thus might not be able to provide meaningful insights into preferences for a quit motivation tool, we also included former smokers in the study. We recruited pulmonologists via email and in person, employing purposive sampling to achieve sample heterogeneity with respect to sex and clinical experience (years in practice). The study was approved by an institutional review board and all participants provided written informed consent.

### Infographic prototype development

#### Infographic content

The infographic was designed as a visual tool which clinicians would review with patients as part of a smoking cessation intervention, not only acting as a prompt for intervention, but also enabling a higher-quality intervention (including pharmacotherapy), and enhancing patient quit motivation [24]. The central message was a display of the difference in FEV1 decline over the next 15 years (estimated reliable time horizon for the prediction equation) with and without smoking cessation. We developed a prototype infographic featuring a simple line graph to maximize the visual impact of this difference over time. Given that percent predicted FEV1 anchors values to a predicted norm (as was effective with a prior static “lung age” infographic [18]), we depicted FEV1 values in the graph as percent predictions rather than volumes.

We then sought to attach clinical descriptors to these FEV1 values in order to demonstrate which patient-relevant morbidities would be averted as a result of the slowing in FEV1 decline after smoking cessation. Given its recognisability to clinicians and the fact that each stage could be attached to evidence-based clinical correlates, we used the GOLD (Global initiative for chronic Obstructive Lung Disease) system to classify lung function impairment in the infographic [3]. Accordingly, the infographic shows that smoking cessation will slow lung function decline, leading to a delay in GOLD stage progression and/or a less advanced projected GOLD stage at a future time point [11, 25]. Through a literature search, we identified which patient-relevant outcomes could be differentiated between GOLD stages, including the following candidate outcomes for display in the infographic: mortality, frequency of COPD exacerbations, healthcare utilization, dyspnea, exercise tolerance, quality of life, mental health, and fatigue [26–33]. We complemented this primarily patient-facing information with a physician-facing, valid, fillable, guided prescription for smoking cessation aid, including bupropion, varenicline, and various doses and formulations of nicotine-replacement therapy (NRT) according to current level of smoking. We added information about smoking cessation resources and prescription facilitators such as reminders of required government drug coverage codes and drug costs.

#### Infographic design

Where applicable, we applied evidence-based best practices for infographic design. These included showing direct comparisons, providing clinical context, progressively modifying pictorial icons to demonstrate transitions, and using symbols and images which would deliver accurate meaning even when interpreted literally [34]. In particular, we avoided a primarily text-based infographic [35], and sought to achieve visual simplicity, while leveraging familiar graphical displays to improve health risk communication to patients with varying levels of health literacy [36].

The prototype was developed with the help of a graphic designer and underwent face validation by a convenience sample of 10 allied health professionals who were each given the prototype for assessment at their convenience and asked to provide unstructured feedback on content and usability. After corresponding improvements were made, we presented the updated prototype to 40 airways disease researchers and clinicians at the Canadian Respiratory Research Network National Meeting [37]. We gathered insights through a moderated full group meeting at this venue, which led to a final round of changes to content and visual elements before launching the rapid-cycle design process.

### Rapid cycle design process

Each “cycle” in the rapid-cycle design process consisted of three sequential stages: (1) infographic testing in a focus group and a clinician interview; (2) review of transcripts for emergent/critical findings; and (3) modifications to the infographic to address findings.

Focus groups included 4–6 patients and lasted one to two hours, whereas interviews lasted one hour. Both focus groups and interviews were facilitated by a trained moderator, attended by a research staff member, audio-recorded, transcribed verbatim, and de-identified. Each session was scripted and utilized a semi-structured format to encourage participants to discuss the infographic on their terms, while still allowing the moderator to probe specific points of inquiry within two pre-set overall themes: infographic content and format. At the end of each session, participants completed questionnaires capturing demographic information and Likert scale-based assessments of infographic content, format, and usage preferences. Focus group participants received $60 in compensation for their time and travel expenses. Pulmonologists additionally completed the Suitability Assessment of Materials (SAM) questionnaire—a systematic method to objectively assess clinicians’ perceived suitability of an infographic for their patients [38]. Raters assessed each of the 22 SAM variables (Table 2) on a scale of 0 (not suitable), 1 (adequate), or 2 (superior), except for the final “cultural appropriateness” variable (an assessment of suitability for one’s population), which was rated on a scale from 0 (not recommended) to 10 (recommended without reservation). The final score is expressed as a mean cumulative score out of 42 (for 21 variables) along with a mean “suitability for population” score out of 10 [38].

### Analysis

In the review of transcripts between sequential cycles, a member of the research team (qualitative analyst) assessed focus group and interview transcripts in detail and grouped relevant quotes into emerging themes within a spreadsheet, while distinguishing between patient (focus group) and physician (interview) quotes. The moderator vetted this spreadsheet to ensure contextual appropriateness of quotes and completeness. The entire research team then reviewed this spreadsheet collaboratively to identify emergent and critical findings. A priori, critical findings were defined as those which all participants in a single focus group or most participants across focus groups/interviews agreed to and/or that the co-principal investigators (co-PIs), analyst, and moderator agreed were likely to be broadly representative, required a change in order to address, and that change was feasible to implement within the infographic. Emergent findings were those that were expressed by more than one participant across a single focus group or across two or more focus groups but which were not felt by co-PIs, the analyst and/or the moderator to be sufficiently important, broadly representative, and/or feasible to implement. Emergent findings could be considered critical after appearance in two or more focus groups or interviews. The pre-set stopping criterion for the study was when no new critical findings emerged from a focus group and interview cycle, thus reaching sufficient information power [39]. If there were divergent opinions during patient focus groups about specific topics, we posed pointed questions to subsequent focus groups until there was a clear directional preference.

At the end of the study, we performed a summative qualitative analysis of all transcripts. Data analysis followed the systematic process prescribed by LeCompte and Schensul, taking place in three stages: (1) item analysis, (2) pattern analysis, and (3) structural analysis [40]. Using the R open source software package for Qualitative Data Analysis (RQDA), we created a coding scheme which enabled us to carry out this inductive analysis by compiling items together at a specific level and then creating more abstract statements about patterns of relationships in the data (themes), to generate overall insights into user preferences for infographic content and format. Consensus on interview coding was reached through comparison and discussion between qualitative analysts (PP, SG, PG), and demonstrative quotations were collected for each identified theme. We also present quantitative summary statistics from demographic, Likert scale, and SAM questionnaires.

## Results

We conducted four sequential cycles of patient focus groups and clinician interviews until stopping criteria were met. The four pulmonologists recruited for interviews (two male, two female) had been in practice for 1–20 years and reported seeing between 2 and 20 COPD patients per week. We recruited 20 COPD patients for focus groups (Table 1).

**Table 1:** Patient background and demographic information (n = 20)

		Number (%)
Sex (n = 19)	Male	11 (58)
	Female	8 (42)
Age (n = 18)	40–49	1 (6)
	50–59	2 (11)
	60–69	6 (33)
	70–79	4 (22)
	≥80	5 (28)
Highest level of education completed (n = 19)	Elementary school	1 (5)
	High school	2 (11)
	College/trade school/other	7 (37)
	University	9 (47)
Total annual personal income (n = 15)	< $20,000	2 (13)
	$20,000–39,999	2 (13)
	$40,000–59,999	3 (20)
	$60,000–79,999	2 (13)
	> $80,000	6 (40)
Time since COPD diagnosis (n = 20)	1–5 years	7 (35)
	6–10 years	6 (30)
	11–15 years	2 (10)
	16–20 years	3 (15)
	≥20 years	2 (10)
Emergency room visits for COPD in the past year	0	16 (80)
(n = 20)	1	3 (15)
	2	1 (5)
Current smoking status (n = 20)	Current smoker	5 (25)
	Prior smoker	15 (75)
Number of prior quit attempts (n = 16)	0–5	9 (56)
	6–10	5 (31)
	11–15	2 (13)

COPD denotes chronic obstructive pulmonary disease. As indicated, the number of subjects who chose to respond to each question varies by question

### Qualitative results

**Fig. 1:**
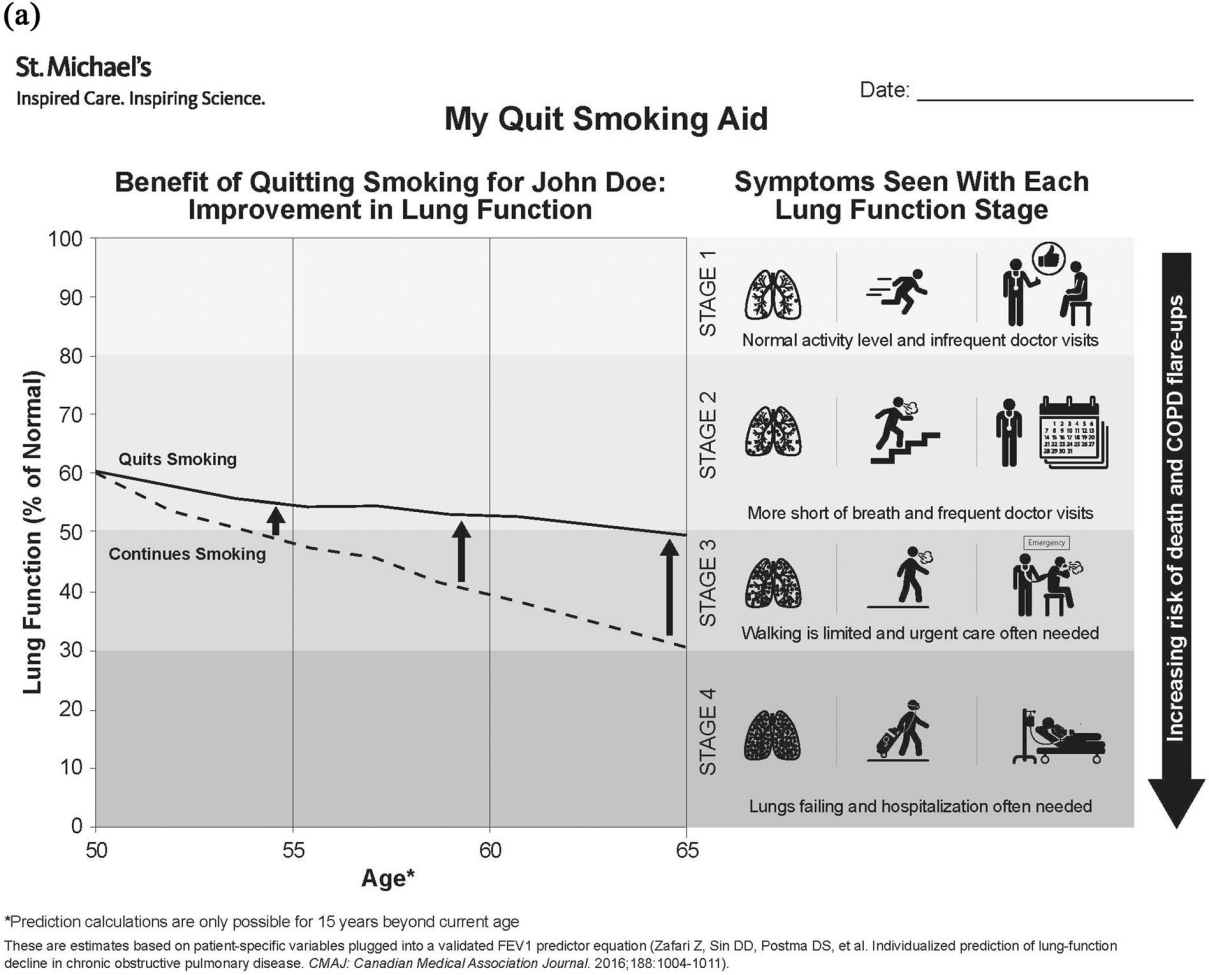

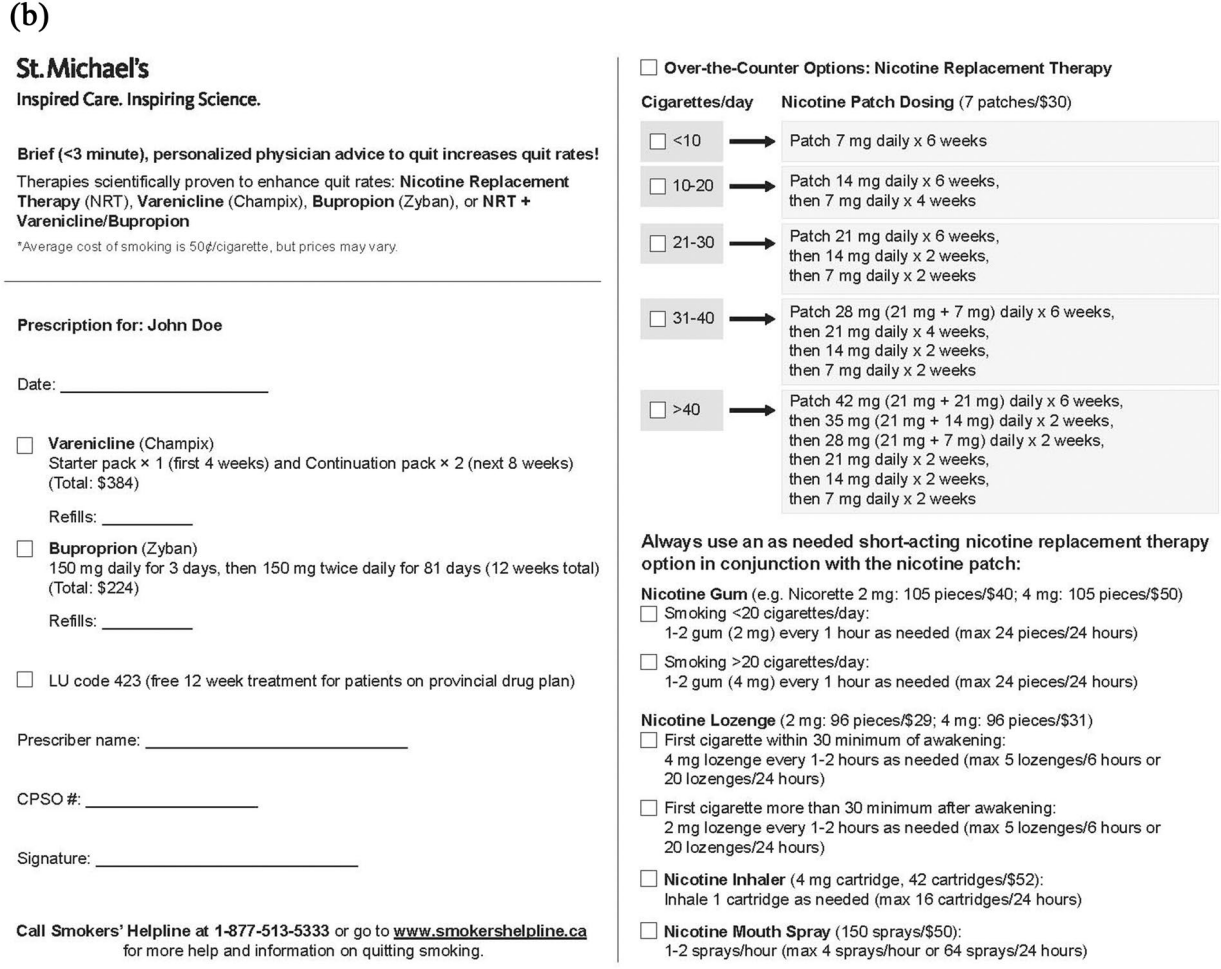
Final infographic. **A** Shows the front page of the infographic, including a line graph demonstrating expected lung function decline with ongoing smoking versus smoking cessation and morbidities associated with each lung function stage (a John Doe infographic is shown for simplicity; a similar Jane Doe infographic is available for female patients). **B** Shows the back page of the infographic, featuring a valid, fillable, guided prescription for smoking cessation aids

Critical findings and corresponding changes made to the infographic and serially updated infographic versions used in each round of the rapid-cycle design process are shown in Additional file 1. The final infographic is shown in Fig. 1.

#### Thematic analysis

Results of the three staged analysis, by each main identified theme and subthemes, are described below. Further representative quotations and the full thematic structure are provided in Table 2.

**Table 2:** Themes with representative quotations from focus groups and interviews

Themes and subthemes	Representative quotations
1. Tool content	
(a) Importance of messaging tone	
Preference for positive messaging	“Yeah, I think the other point is that if we’re using this to—as a tool to urge people to quit, we want to keep the message as positive as possible. You don’t want too much fear in it, the down line plus the graphics there, you know, might be a little too negative” (R2, P2)
Preference for negative messaging	“I think it would even be maybe more effective to say, increasing risk of COPD flares and death. Because again, most patients are probably going to be, in the visceral sense, more aware of what it’s like to have a COPD flare than the thought of their mortality.” (R1, C1) “I think it’s more meaningful if [the arrow beside the graph points] down […] This is where you’re going to end up.” (R2, P2) “It’s scarier if the arrows are going down.” (R2, P3)
(b) Importance of Personalization	
	“…the fact that it’s personalized to the patient is very, very powerful, and it’s readily available. And then they leave with that in their hand, where it’s a story about them. I think that’s a very powerful thing.” (R3, C3) “Because this has happened to me, stage three, instead of puffing when you’re walking, use a walker, have a rollator there” (R3, P1)
(c) Importance of Lay Language	
	“What falls under COPD? Is that, like, asthma? Just any chronic obstructive pulmonary disorder? […] Yeah, I’ve heard emphysema and chronic bronchitis, but I didn’t know if asthma was classified as COPD.” (R1, P4)
(d) Scientific vs Intuitive Display Preference	
Preference for scientific display (physicians)	“Those look like black dots. That looks like you’re developing some sort of fibro nodular disease. So I don’t know, I think you need a better graphic ‘cause that’s not what lungs that smoke look like. You don’t have fibro nodular disease, you have holes, right, and you’re not showing that.” (R2, C2)
Preference for intuitive display (patients)	“Yeah, you see the bronchia, which, you know, in the others, it’s more congested. You can’t see it. So that I got.” (R1, P1) “Just flop them so your darker lung is at the bottom.” (R1, P3)
2. Tool Design	
Importance of engaging design	“Yeah, because this—the graph and the way it’s set up is—it’s kind of blah, frankly. You want something to grab you there. This is—like, I mean, it’s like a—one of those Rothko paintings. It’s just white with a little dot or something, you know. It’s not grabby enough.” (R1, P2) “There is no focal point on this. Usually with an infographic, you’re supposed to have a focal point that draws you in. There is no focal point here.” (R2, C2)
Preference for minimalist design	“You make me—when you ask me what I’m taking home from it, essentially I’m taking home the whole thing, the whole top. If there was too much information on it […] [it would] be in overload […] So yeah, I think it’s very effective in that way,that it doesn’t go overboard. Doesn’t give you too much info. But it gives you enough to think about.” (R1, P2) “It’s really busy. It’s too much stuff. That’s my first thought.” (R2, C2)
Preference for large fonts and visual elements	“Print’s too small.” (R1, P5) “It is way too small.” (R1, P3) “And it’s too crowded.” (R1, P5) “It’s too small […] The problem for me right now is in black and white, I’m not being drawn to the key message. I want to see these two lines, you know, Jane quit smoking, Jane continues smoking, those should be bigger.” (R2, C2)
Preferences for use of colours	“I would say something that’s more colourful.” (R1, P4) “It’s true, we are very visual, you know what I mean, and red, danger and certain things like that, right.” (R1, P3) “I think that this being in colour, even though it’s being more expensive, I think that would be really nice, especially for the graph parts of the– it’s just like a much more clearer relation of the stages, you know, just to colour code it.” (R4, C4)
3. Advantages of Infographic Messaging	
(a) Overall power of visual depictions	Yeah, [if this was] tied to the lung function test? Absolutely, this is a visual of what that is in layman’s terms.” (R2, P4)
(b) Function of anchoring to evidence	“This is objective evidence. So I think that would be very helpful in that regard.” (R2, C2) “…everything that’s been recommended here has been validated and shown to be helpful in some patient populations.…for the doctor to be able to say, we know these work.” (R2, C2)
(c) Accessibility to low literacy and non-English speaking populations	“A lot of my patients will take notes, because many of them don’t speak English…So for my population, where I would be using this, I prefer something where they’ve got a pen and they can scribble all over it.” (R2, C2)
4. Impact of Tool on Determinants of Smoking Cessation Advice Delivery	
(a) Impact on barriers to smoking cessation advice delivery	
Tool acts as a reminder to deliver smoking cessation advice	“And it’s a reminder, right, so it’s almost like you’ve done a PFT, COPD, this is on there. It’s like a constant reminder to also talk to those patients that you’ve seen time and time and time again” (R4, C4)
Tool increases efficiency of smoking cessation advice delivery	“I think not only would you be faster. I think you would be more organized. And I think over time you would actually see even, like, additive benefits of that because you would get more comfortable providing smoking cessation counselling in an organized manner. And in a way that leads you directly from the explaining the relevance to the patient and then kind of going right into the therapeutic options. I think it would be really quick and efficient.” (R1, C1)
Tool increases impact of smoking cessation advice intervention	“…the fact that the patient would then get this and be able to reference it back and maybe they would stick it on their fridge or have it somewhere where they could look at it and it would continue to motivate them at home after their healthcare visit. And might even make them want to do some of their own reading about the effects of smoking” (R1, C1)
Effect on relationship with patient (deleterious impact)	“So if someone says I’m not interested in quitting smoking, and then I go on and say, okay John, you told me you don’t want to quit smoking. But let’s talk more about your lungs and what’s going to happen. They’re just going to say, listen, this guy is not hearing me. I told him I don’t want to quit.” (R3, C3)
Effect on relationship with patient (positive impact)	“…they want information, and this is information. They may not do anything with it, but they really appreciate, they really appreciate somebody sitting down and talking to them.” (R2, C2)
(b) Enabling impact on smoking cessation advice delivery	
Ease of access	“… it makes it much easier than having to find something else and print it off and see if you’re in a room that has any posters or pictures. I like tools like this. This is very useful ‘cause I find just ease of access, it’s right there.” (R4, C4)
Prescription facilitation: reminder of medication doses	“I think it would be helpful. It’s always—you know, it’s just to remember exactly the right dose of nicotine replacement therapy given your patient’s smoking habits.” (R1, C1)
Prescription facilitation: reminder of medication contraindications	“I don’t do a ton of COPD care as respirologist myself…I would want more information for…the Champix and Zyban. Just at least what to avoid, right.” (R4, C4)
Prescription facilitation: reminder of medication coverage	“… I think it’s helpful that it has the costs incorporated as well, which are an important piece because they’re not always covered unless you’re in one of these kind of special referral programs…” (R1, C1)
Role of physician guidance	“I think one of the big advantages of it is that the little graphics, the pictures here, the doctor can actually point to them and say, look, how short of breath are you on a scale of one to ten, or something…The doctor can give you what to look for in stages one to ten by pointing to a particular activity” (R1, P2)
5. Barriers and enablers to quitting	
(a) Barriers to quitting	
Financial	“But even on the left here, Chantix all that, $384 for 8 weeks of (R4, P2)—“Who’s got that kind of money?…They want you to quit, but it’s so expensive to quit.” (R4, P5)
Fear of mediation side-effects/dependency	“…I broke out. I did, you know, right arm, left arm, chest, the whole bit to the point where I had to go to a dermatologist. I tried another couple of patches and still had a problem…” (R2, P2)
Perceived futility of quitting when predicted GOLD stage does not change with smoking cessation	“So in the scenario where you may end up in the same stage whether or not you quit, do you think that you’d still be compelled to quit smoking if you saw that? (R2, M) “I’d be—yeah, number two, less compelled.” (R4, P2) “Yeah, I wouldn’t quit.” (R4, P6)
Patient not being in the Active Stage of Quitting	“Maybe I can quit. But it took a long time. It took two years to get myself to a spot where I can honestly say I quit smoking.” (R2, P2)
(b) Enablers to quitting	
Fear of worsening functional capacity	“Homecare that you’d need. Possibility of being in a wheelchair or having to use oxygen tanks. Just, you know what, losing your ability to be independent is terrifying.” (R1, P3) “I find the fear of my patients who have COPD are smoking, are petrified of being on oxygen…” (R3, C3)
Fear of worsening lung function	“I think the lungs because you feel it. You feel that your lung’s not—hasn’t got the capacity.” (R3, P5) “I could turn around a lot of people who are in stage one and open their eyes as to what stage two is, because…here’s stage two and it creeps up on you. And that’s the thing about the disease, you really don’t notice it. It just creeps up on you.” (R3, P2)
Fear of frequent flare-ups	“That’s not something that you can just get a script in the emerge and you’re okay…It’s not just a simple tablet you take once in awhile when you show up in the emergency department. Yes, you do for a flare but it shouldn’t be that that’s the message they’re getting. It should be, like, you’re ending up in the emerge a lot and you’re sick, you know.” (R4, C4)
Fear of death	“…The mention of death is more effective.” (R4, P4)
Physical depictions of lung deterioration	“… Just black out that part of the lung that’s gone dead, if you like… a picture would show only a third of a lung in clear and the rest of it’s black or greyed out…I just think it would be more dramatic, be more impactful…” (R3, P5)
Diverse smoking cessation pharmacotherapy options and resources	“Oh, smokers help line…in fact, I would put that at the top before anything to know that there was some place that you could definitely go.” (R2, P1)

C denotes clinician participant; M denotes moderator; P denotes patient participant; R denotes round

#### Tool content

The first theme included all preferences and observations pertaining to the content within the infographic. The first Content subtheme was the *Importance of Messaging Tone,* wherein some participants believed that a negative overall tone would be more impactful, whereas others preferred a positive tone: “…if we’re using this to—as a tool to urge people to quit, we want to keep the message as positive as possible” [Round 1 (R1), Patient 2 (P2)]. Participants stressed the *Importance of Personalization* of infographic content in order to ensure patient buy-in: “…the fact that it’s personalized to the patient is very, very powerful… it’s a story about them. I think that’s a very powerful thing.” [R3, Clinician 3 (C3)]. In addition, participants identified the *Importance of Lay Language* to prevent confusion: “[COPD is] either emphysema or chronic bronchial disease. It’s one of the two.” (R2, P4). The last Content subtheme was about a *Scientific vs Intuitive Display Preference*, wherein clinicians indicated a clear preference for scientifically accurate depictions of disease, such as lung damage in COPD: “…it’s just going to look like there’s a lot of black dots, which would be okay ‘cause it means there’s a lot of carbon deposition. But that’s not what you’re trying to show” (R2, C2). Conversely, patients preferred more intuitive illustrations of lung damage: “Just black out that part of the lung that’s gone dead, if you like… a picture would show only a third of a lung in clear and the rest of it’s black or greyed out.” (R3, P5).

#### Tool design

The second theme encompassed design preferences for the infographic. Design features included: *Importance of Engaging Design (*“I just wanted something that would sort of jump out at you, you know.”) (R1, P2); a *Preference for Minimalist Design* (“The problem is then you have so much stuff […] there might be an opportunity to lay it out a bit different”) (R3, C3); a *Preference For Larger Fonts And Visual Elements* (“…it would have much more impact if it were larger”) (R1, P5); and a *Preference For Use Of Colours (*“Yeah, I would do all the graphics in different colours there, not just the lungs.”) (R1, P3).

#### Advantages of infographic messaging

The third theme identified how using an infographic in such a tool could confer specific benefits over other approaches. The first associated subtheme was *Overall Power of Visual Depictions*, wherein participants indicated a preference for visual communication for conveying complex information: “Just on the face of the document, my first impression is that I can—there’s a lot of information here and I can gather it at a glance” (R3, P3). The second subtheme was *Function of Anchoring to Evidence*, wherein participants described the value of communicating the scientific basis of infographic messages: “But certainly having the reference down here to actually justify the decreased lung function prediction, I think is really useful. Especially in the subset of patients that have more higher-level questions about where this information’s coming from” (R1, C1). Finally, participants stressed the advantage of using an infographic approach as a means to improve *Accessibility to Low Literacy and Non-English Speaking Populations*: “There’s not a lot of writing, so I find that more useful ‘cause people are usually sick of reading things…and not everybody has the same literacy level…or speaks English, you know” (R4, C4).

#### Impact of tool on determinants of smoking cessation advice delivery

The fourth theme featured participants’ impressions of the impact of this tool on the barriers and enablers to delivery of smoking cessation advice in clinical practice. The first subtheme was the *Impact on barriers to smoking cessation advice delivery*, wherein participants discussed the effect of using the tool on patient-physician interactions and the therapeutic relationship. For example, clinicians discussed how the tool might not only act as a reminder for them to deliver smoking cessation advice, but could also overcome perceived lack of effectiveness of that advice: “It’s like a constant reminder to also talk to those patients that you’ve seen time and time and time again” (R4, C4); “I feel like it would just add to how people take in information […] I think it would enhance that for some people” (R4, C4). The second subtheme was about the tool’s *Enabling impact on smoking cessation advice delivery*, such as how ease of access to smoking cessation information through a printed copy of this tool could facilitate information delivery (“… I think if I could open a drawer, pull up a smoking cessation tool and kind of counselling about COPD at the same time, that would be fantastic”) (R3, C3); and the fact that the infographic simplifies smoking cessation prescriptions by listing doses, contraindications, and medication coverages.

#### Barriers and enablers to quitting

The final theme elucidated participants’ awareness of barriers and enablers to quitting smoking, and how these were addressed in the infographic. The first subtheme was *Barriers to Quitting*, including: the cost of smoking cessation aids (“I’m on disability and so, like, [nicotine replacement therapy is] not something that I can afford to buy”) (R1, P3); concerns about tolerability of smoking cessation aids (“I went on was Champix and I was deathly ill over it. […] Deathly, deathly ill.”) (R2, P2); perceived futility of quitting when smoking cessation did not lead to a change in predicted GOLD stage in the infographic (“Yeah, there’s no advantage…Absolutely it’s less compelling”) (R3, P1); and unreadiness to actively engage in quitting (“You have to be ready to quit or you won’t quit. You got to want to quit”) (R3, P4) (a concern which guidelines have debunked [6]).

The second subtheme in this category was *Enablers to Quitting*, including fears of: worsening functional capacity (“I thought, am I going to give up sports? I don’t think so. So about a month later—and it bothered me so bad I quit”) (R1, P3); worsening lung function (“I think if you seen one in six months and then you seen one in a year and it showed a decrease in your lung function and you went, let’s say from a stage two to a stage three, that would probably scare somebody.”) (R4, P6); frequent flare-ups (“Yeah, death but also the COPD flare-ups, because that’s an indicator of where you’re going.”) (R4, P1); and death (“Keep it straight. If you keep smoking you’re going to die. And I think it’s a very powerful message”) (R2, C2). Other enablers included the power of physical depictions of lung deterioration (“I just think it would be more dramatic, be more impactful that’s all. Whoa, what do you mean? Less than half my lungs are active? That’s the way I would read it.”) (R3, P5) and the availability of diverse smoking cessation options and resources in the infographic (“I think it’s good that you’re showing other alternatives to the patch, so it’s not—you know what I mean. Like, you’re not alone. There’s gum and there’s spray. I think this is a good list”) (R1, P4).

### Quantitative results

#### Patients

**Fig. 2:**
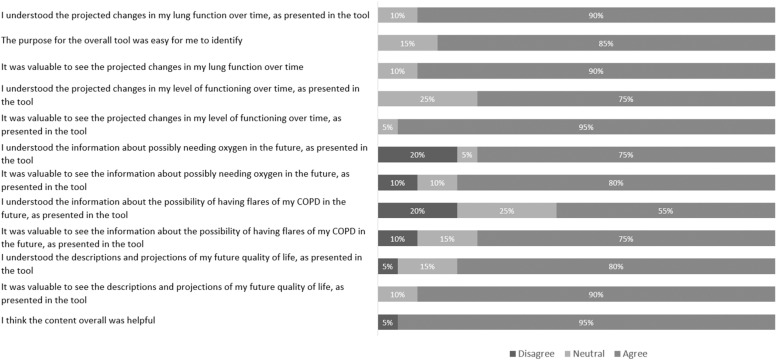
Summative Patient Feedback: Infographic Content. Responses were entered on a five-point Likert scale labeled 1 (disagree), 3 (neutral), and 5 (agree). In this figure, scores of 1 and 2 were considered “disagree,” and scores of 4 and 5 were deemed “agree.” Each bar demonstrates the proportion of patients with each response, for each statement

**Fig. 3:**
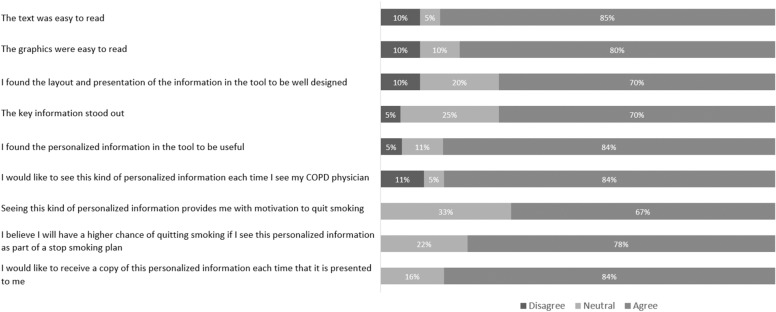
Summative Patient Feedback: Infographic Design, Usage, and Impact. Responses were entered on a five-point Likert scale labeled 1 (disagree), 3 (neutral), and 5 (agree). In this figure, scores of 1 and 2 were considered “disagree,” and scores of 4 and 5 were deemed “agree.” Each bar demonstrates the proportion of patients with each response, for each statement

Likert-scale response values are presented in summative fashion for all four focus groups (Figs. 2, 3). Overall, there was a trend to more favorable responses as the study progressed, with improvement in the mean Likert scale response score for 11/12 content-related questions (improvement from a mean of 3.8/5 in the first focus group to 4.5/5 in the last focus group), 4/4 design-related questions (improvement from a mean of 3.2 in the first focus group to 4.2 in the last focus group), and 5/5 tool usage and impact-related questions (mean improvement from a mean of 4.0 in the first focus group to 4.8 in the last focus group).

#### Clinicians

In exit questionnaires, all four physicians indicated that if the printout were made available, they would look at it all or most of the time and would discuss it with their patient all or most of the time. All physicians felt confident in their ability to present the information in the tool to their patients and all believed that it would be important to be able to provide a copy to patients. Two of four physicians believed that the tool would help their patients to quit smoking (the other two were neutral), and three of four indicated that they would use the tool as a prescription.

The mean overall SAM score was 77.8% (32.7/42), corresponding to a “superior” overall rating [SAM percentages are grouped as follows: 0–39% (not suitable), 40–69% (adequate), and 70–100% (superior) [41]]. Clinicians rated the suitability of the infographic for their population as 8.75/10. Mean scores for each SAM variable are reported in Table 3.

**Table 3:** Clinician suitability assessment of materials (SAM) instrument scores

SAM area	SAM variable	Mean score (/2)^a^
Content	Purpose	1.75
	Content topics	1.75
	Summary and review	1.66
Literacy demand	Reading grade level	1.25
	Writing style	1.75
	Sentence construction	1.75
	Vocabulary	1.75
	Learning enhanced by advance organizers	1.50
Graphics	Cover graphic	1.50
	Type of illustrations	1.25
	Relevance of illustrations	1.50
	Graphics: lists, tables, charts, forms	1.50
	Captions are used to “announce” or explain graphics	1.75
Layout and typography	Typography	1.00
	Layout	1.50
	Subheadings and “chunking”	1.75
Learning stimulation and motivation	Interaction included in text and/or graphics	1.00
	Desired behavior patterns are modeled or shown in specific terms	1.75
	Motivation	1.50
Cultural appropriateness	Cultural match—logic, language, experience (LLE)	1.66
	Cultural Image and Examples	1.33
	Suitability for population^b^	8.75/10

^a^A mean of individual responses from each clinician, entered on a three-point scale for each SAM variable (0 = not suitable; 1 = adequate; 2 = superior)

^b^Clinicians were asked to rate suitability based on patient socioeconomic/cultural backgrounds on a scale of 1–10

## Discussion

Through an iterative design process with patient and clinician input, we designed a usable and accessible personalized risk assessment tool to drive and facilitate delivery of smoking cessation counselling to patients with COPD.

Our multi-staged and evidence-based design process ensured that the final tool is an accurate reflection of user preferences. We built the infographic around a validated FEV1 decline calculator [11], anchoring FEV1 to the GOLD severity classification system to enable depiction of evidence-based patient-relevant morbidities with disease progression through each stage [3]. In developing our prototype, we espoused best practices for infographic design [34]. In iterating infographic content and design, we then employed an integrated knowledge translation process [42], engaging end-users in each step, as has shown to be most effective for later uptake [34].

Rapid-cycle design has been shown to facilitate problem exploration, enhance patient engagement, and improve tentative solutions through incremental analysis [23]. There were evident improvements in scores across content, design, and impact of the infographic from its first to last iteration in the rapid-cycle process, in keeping with serial improvement. At the same time, summative scores across these domains were highly favorable. Subject agreement with questions about comprehensibility and value of infographic content elements was ≥ 75% for 11/12 questions, and 95% of subjects agreed that the content was overall helpful (Fig. 2). We do note that only 55% of subjects agreed with a statement that they understood their risk of future COPD exacerbations through the infographic, and this is an example of an outcome that would likely need further clarification through interaction with the clinician. Feedback on infographic usage and impact was positive, with 84% of subjects indicating a desire to see this kind of tool at every visit and 78% believing that it would increase their chances of quitting (Fig. 3). Similarly, all clinicians indicated that they would use the tool with their patients, stating that they were confident in their ability to present the information in the tool.

Because biomedical risk is a complex concept to communicate with patients and ensuring patient relevance is critical to driving quit motivation, we chose to communicate risk through an infographic [15]. Use of infographics for risk communication has been shown to improve health information uptake and may improve patients’ decision-making capacity, including for smoking cessation [19, 43–45]. Infographics are also particularly well suited to support comprehension among individuals with low health literacy. We used the validated SAM metric because it has been shown to predict likelihood of uptake of health educational materials, particularly in low-education, low-literacy patient populations [34, 46]. Our approach enabled us to achieve a SAM score of 77.8%, corresponding to a “superior” overall rating [41].

Previous studies report several forms of biomedical risk assessment in people who smoke (i.e. providing feedback on the physical effects of smoking using physiological measurements), though their impact has not been assessed in patients with COPD specifically. Parkes, et al. showed a significant absolute increase in cessation rates of 7.2% by providing immediate feedback and explanation of spirometry results in the form of a patient-specific “lung age,” compared to the smoker’s chronological age [18]. Our infographic performs as an “enhanced” lung age calculator, by providing not only a static calculation of relative lung function impairment, but also a predictor of *future* lung function and corresponding expected future patient-relevant morbidities.

Although biomedical risk assessments do not consistently improve smoking cessation rates when provided in isolation and without a theoretical foundation for behaviour change [15, 47], it is our association of biomedical risk with specific patient-relevant outcomes that we believe can impact quit motivation [24]. At the same time, the infographic acts as a provider prompt for a brief smoking cessation intervention (and may improve the quality of that intervention), which in itself is proven to improve quit rates [7]. Accordingly, our tool leverages several theory-based behaviour change strategies, targeting both patient-level determinants (barriers and enablers) of cessation and clinician-level determinants of providing smoking cessation advice and pharmacotherapy. Findings from our qualitative analysis support this theoretical framework and compliment existing literatures in the areas of personalized biomedical risk assessment design and implementation, and determinants of smoking cessation intervention success. For example, in applying Michie’s behaviour change framework [48], Aumann and colleagues identified provision of information about the consequences of smoking in a visual and a “drastic” form as strong quit motivators [24]. Patients in our study expressed a similar belief in the “power of visual depictions” and the motivation provided by a fear of worsening and “scarier” messaging, including intuitive physical depictions of lung damage. On the contrary, some patients believed that a positive tone would be more effective than a negative one. This reflects a similar divergence in the literature, whereby some authors, citing prospect theory, suggest that “gain-framed statements” (emphasizing the benefits of quitting) are more effective than “loss-framed statements” [49]. This divergence in preferences, and the corresponding finding that about one third of participants were neutral when asked if the infographic provided motivation to quit (Fig. 3), suggests that personalizing overall tone may be required for maximal effect. On the clinician side, the infographic itself was believed to act as a reminder for, and a means to increase the efficiency and perceived impact of smoking cessation advice delivery. These effects address known provider-level barriers, including forgetting to deliver advice, having insufficient time, and not believing that advice would be effective enough to motivate a quit (“outcome expectancy”) [50, 51]. Patients and clinicians also indicated a strong preference for personalization within the infographic. Indeed, tailored and personalized feedback has previously been shown to augment the effectiveness of physician-delivered smoking cessation interventions, through more effective quit motivation [21, 43, 44, 52], and personalization of advice is recommended in guidelines [7, 21]. Finally, it is of note that some providers indicated a concern about potential deleterious impacts on the patient-physician relationship through delivery of advice. Previous research has also identified this concern as the strongest attitudinal predictor of advice delivery [53]. It is encouraging, however, that some providers believed that a patient-facing infographic might engender a more positive patient interaction.

Our study has several limitations. Although we recruited a diverse patient population with respect to sex, income, age, quit attempts, and COPD duration, the rapid cycle design process involved a small sample size of four clinicians and 20 patients. Furthermore, 25% of the patients were current smokers and 75% were former smokers, whereby most participants provided feedback based on their recollection of motivators for smoking cessation (subject to recall bias) rather than their current experience, representing a threat to generalizability of our findings to all current smokers. We also acknowledge that only 14% of participants had a high-school education or less, further threatening generalizability to lower literacy and more marginalized populations. However, SAM results support accessibility across various health literacy levels and the tool is designed to be used at the point-of-care, with clinician guidance rather than by patients alone. Finally, our study did not include primary care physicians, and future studies should aim to determine whether their preferences differ, given their critical role in smoking cessation counselling.

## Conclusions

In summary, we used an iterative rapid-cycle design process to develop and optimize a patient- and clinician-facing personalized biomedical risk assessment tool predicting future lung function decline, to drive smoking cessation. Our findings suggest that this process led to a tool that reflects user preferences, would be usable in real-world clinical settings, and impacts behavioural determinants of provider smoking-cessation advice delivery, while increasing patient quit motivation. To enable this personalized infographic to be provided to each patient after pulmonary function testing, we are now developing an algorithm-based software that interacts with pulmonary function software at the point-of-care to automatically generate a personalized infographic. Next, we will explore and measure the impacts of this system on provider delivery of smoking cessation advice and pharmacotherapy prescription, patient motivation to quit, and smoking cessation success in real-world clinical settings.

## Supplementary Information

**Additional file 1**: Serial changes to infographic in rapid-cycle design process. John Doe infographic is shown for simplicity; a similar Jane Doe infographic was also assessed. (A) 1-page infographic used in focus group 1, user comments and corresponding changes made. (B) 2-page infographic used in focus group 2, user comments and corresponding changes made. (C) 2-page infographic used in focus group 3, user comments and corresponding changes made. (D) 2-page infographic used in focus group 4, user comments and corresponding changes made.

## Data Availability

The datasets used and/or analysed during the current study are available from the corresponding author on reasonable request.

## Abbreviations

COPD: Chronic obstructive pulmonary disease
FEV1/FVC: Forced expiratory volume in one second/forced vital capacity ratio
GOLD: Global initiative for chronic obstructive lung disease
NRT: Nicotine-replacement therapy
SAM: Suitability assessment of materials
Co-PI: Co-principal investigators
RQDA: R open source software package for qualitative data analysis
LLE: Logic, language, experience

## References

[1] World Health Organization. Chronic obstructive pulmonary disease (COPD): World Health Organization; 2017. Available from: https://www.who.int/news-room/fact-sheets/detail/chronic-obstructive-pulmonary-disease-(copd.

[2] López-Campos J, Tan W, Soriano J (2016). Global burden of COPD. Respirology.

[3] Global Initiative for Chronic Obstructive Lung Disease. Global strategy for the diagnosis, management, and prevention of chronic obstructive pulmonary disease 2021 report. Global Initiative for Chronic Obstructive Lung Disease 2021.

[4] Wheaton A, Cunningham T, Ford E, Croft J. Employment and activity limitations among adults with chronic obstructive pulmonary disease—United States, 2013. MMWR Morb Mortal Wkly Rep. 2015;64(11). PMC4584881 25811677

[5] Stead L, Bergson G, Lancaster T. Physician advice for smoking cessation. The Cochrane database of systematic reviews. 2008(2). 10.1002/14651858.CD000165.pub3 18425860

[6] Leone F, Zhang Y, Evers-Casey S, Evins A, Eakin M, Fathi J (2020). Initiating pharmacologic treatment in tobacco-dependent adults. An Official American Thoracic Society Clinical Practice Guideline. Am J Respir Crit Care Med.

[7] Krist A, Davidson K, Mangione C, Barry M, Cabana M, Caughey A (2021). Interventions for tobacco smoking cessation in adults, including pregnant persons: US Preventive Services Task Force Recommendation Statement. JAMA.

[8] Schuster M, McGlynn E, Brook R (1998). How good is the quality of health care in the United States?. Milbank Quart.

[9] Statistics Canada. Canadian Tobacco Use Monitoring Survey (CTUMS) Canada: Statistics Canada; 2007 Available from: https://www23.statcan.gc.ca/imdb/p2SV.pl?Function=getSurvey&Id=30140.

[10] Fletcher C, Peto R (1977). The natural history of chronic airflow obstruction. Br Med J.

[11] Zafari Z, Sin D, Postma D, Löfdahl C, Vonk J, Bryan S, et al. Individualized prediction of lung-function decline in chronic obstructive pulmonary disease. CMAJ Can Med Assoc J. 2016;188(14). 10.1503/cmaj.151483 PMC5047815 27486205

[12] Glazebrook C, Garrud P, Avery A, Coupland C, Williams H. Impact of a multimedia intervention “Skinsafe” on patients’ knowledge and protective behaviors. Prevent Med. 2006;42(6). 10.1016/j.ypmed.2006.02.007 16580059

[13] Glanz K, Steffen A, Schoenfeld E, Tappe K. Randomized trial of tailored skin cancer prevention for children: the Project SCAPE family study. J Health Commun. 2013;18(11). 10.1080/10810730.2013.778361 PMC3815976 23806094

[14] Marteau T, French D, Griffin S, Prevost A, Sutton S, Watkinson C, et al. Effects of communicating DNA-based disease risk estimates on risk-reducing behaviours. Cochrane Database Syst Rev. 2010(10). 10.1002/14651858.CD007275.pub2 PMC12853416 20927756

[15] Bize R, Burnand B, Mueller Y, Rège WM, Cornuz J. Biomedical risk assessment as an aid for smoking cessation. Cochrane Database Syst Rev. 2009(2). 10.1002/14651858.CD004705.pub3 19370604

[16] Bovet P, Perret F, Cornuz J, Quilindo J, Paccaud F (2002). Improved smoking cessation in smokers given ultrasound photographs of their own atherosclerotic plaques. Prevent Med..

[17] Takagi H, Morio Y, Ishiwata T, Shimada K, Kume A, Miura K (2017). Effect of telling patients their “spirometric-lung-age” on smoking cessation in Japanese smokers. J Thorac Dis.

[18] Parkes G, Greenhalgh T, Griffin M, Dent R (2008). Effect on smoking quit rate of telling patients their lung age: the Step2quit randomised controlled trial. BMJ (Clin Res Ed)..

[19] McCrorie A, Donnelly C, McGlade K. Infographics: healthcare communication for the digital Age. Ulster Med J. 2016;85(2). PMC4920488 27601757

[20] Graham I, Logan J, Harrison M, Straus S, Tetroe J, Caswell W (2006). Lost in knowledge translation: time for a map?. J Contin Educ Health Profess..

[21] Roberts N, Kerr S, Smith S (2013). Behavioral interventions associated with smoking cessation in the treatment of tobacco use. Health Serv Insights..

[22] Kitzinger J (1995). Qualitative research. Introducing focus groups. BMJ (Clin Res Ed).

[23] Johnson K, Gustafson D, Ewigman B, Provost L, Roper R. Using rapid-cycle research to r each goals: awareness, assessment, adaptation, acceleration. Rockville, MD: Agency for Healthcare Research and Quality; 2015.

[24] Aumann I, Tedja L, von der Schulenburg J. Experiences of COPD patients with existing smoking cessation programs and their preferences for improvement—a qualitative analysis. Tob Induc Dis. 2016;14(1). 10.1186/s12971-016-0097-4 PMC4997659 27563285

[25] Jones P, Brusselle G, Dal Negro R, Ferrer M, Kardos P, Levy M (2011). Properties of the COPD assessment test in a cross-sectional European study. Eur Respir J.

[26] Dusser D, Wise R, Dahl R, Anzueto A, Carter K, Fowler A (2016). Differences in outcomes between GOLD groups in patients with COPD in the TIOSPIR(®) trial. Int J Chronic Obstr Pulm Dis..

[27] Hurst J, Vestbo J, Anzueto A, Locantore N, Müllerova H, Tal-Singer R (2010). Susceptibility to exacerbation in chronic obstructive pulmonary disease. N Engl J Med.

[28] Wallace A, Kaila S, Bayer V, Shaikh A, Shinde M, Willey V (2019). Health care resource utilization and exacerbation rates in patients with COPD stratified by disease severity in a commercially insured population. J Manag Care Specialty Pharm..

[29] Chhabra S, Gupta A, Khuma M (2009). Evaluation of three scales of dyspnea in chronic obstructive pulmonary disease. Ann Thorac Med..

[30] Albuquerque A, Nery L, Villaça D, Machado T, Oliveira C, Paes A (2006). Inspiratory fraction and exercise impairment in COPD patients GOLD stages II–III. Eur Respir J.

[31] Ståhl E, Lindberg A, Jansson S, Rönmark E, Svensson K, Andersson F, et al. Health-related quality of life is related to COPD disease severity. Health Qual Life Outcomes. 2005;3. 10.1186/1477-7525-3-56 PMC1215504 16153294

[32] Janson C, Marks G, Buist S, Gnatiuc L, Gislason T, McBurnie M (2013). The impact of COPD on health status: findings from the BOLD study. Eur Respir J.

[33] Lewko A, Bidgood P, Garrod R. Evaluation of psychological and physiological predictors of fatigue in patients with COPD. BMC Pulm Med. 2009;9. 10.1186/1471-2466-9-47 PMC2774290 19845947

[34] Arcia A, Suero-Tejeda N, Bales M, Merrill J, Yoon S, Woollen J (2016). Sometimes more is more: iterative participatory design of infographics for engagement of community members with varying levels of health literacy. J Am Med Informat Assoc..

[35] Balkac M, Ergun E (2018). Role of Infographics in Healthcare. Chin Med J.

[36] Ancker J, Senathirajah Y, Kukafka R, Starren J. Design features of graphs in health risk communication: a systematic review. J Am Med Informat Assoc. 2006;13(6). 10.1197/jamia.M2115 PMC1656964 16929039

[37] Canadian Respiratory Research Network. Research And Publications: Canadian Respiratory Research Network; 2021. Available from: https://respiratoryresearchnetwork.ca/research-platforms.

[38] Doak C, Doak L, Root J. Teaching patients with low literacy skills. 2nd edn. Philadelphia, PA: Lippincott; 1996.

[39] Malterud K, Siersma V, Guassora A. Sample size in qualitative interview studies: guided by information power. Qual Health Res. 2016;26(13). 10.1177/1049732315617444 26613970

[40] LeCompte M, Schensul J (1999). Designing and conducting ethnographic research.

[41] Wallace L, Rogers E, Turner L, Keenum A, Weiss B (2006). Suitability of written supplemental materials available on the Internet for nonprescription medications. Am J Health-Syst Pharm..

[42] Canadian Institute of Health Research. Guide to Knowledge Translation Planning at CIHR: Integrated and End-of-Grant Approaches Canada: Canadian Institute of Health Research; 2015. Available from: https://cihr-irsc.gc.ca/e/45321.html#f1.

[43] Voncken-Brewster V, Tange H, de Vries H, Nagykaldi Z, Winkens B, van der Weijden T (2015). A randomized controlled trial evaluating the effectiveness of a web-based, computer-tailored self-management intervention for people with or at risk for COPD. Int J Chronic Obstruc Pulm Dis..

[44] Strecher V (1999). Computer-tailored smoking cessation materials: a review and discussion. Patient Educ Counsel..

[45] Jonsdottir H (2013). Self-management programmes for people living with chronic obstructive pulmonary disease: a call for a reconceptualisation. J Clin Nurs.

[46] Ryan L, Logsdon M, McGill S, Stikes R, Senior B, Helinger B (2014). Evaluation of printed health education materials for use by low-education families. J Nurs Scholarsh.

[47] French D, Cameron E, Benton J, Deaton C, Harvie M (2017). Can communicating personalised disease risk promote healthy behaviour change? A systematic review of systematic reviews. Ann Behav Med.

[48] Michie S, van Stralen M, West R. The behaviour change wheel: a new method for characterising and designing behaviour change interventions. Implement Sci. 2011;6. 10.1186/1748-5908-6-42 PMC3096582 21513547

[49] Toll B, Rojewski A, Duncan L, Latimer-Cheung A, Fucito L, Boyer J (2014). “Quitting smoking will benefit your health”: the evolution of clinician messaging to encourage tobacco cessation. Clin Cancer Res.

[50] Young J, Ward J (2001). Implementing guidelines for smoking cessation advice in Australian general practice: opinions, current practices, readiness to change and perceived barriers. Fam Pract.

[51] Sharpe T, Alsahlanee A, Ward K, Doyle F (2018). Systematic review of clinician-reported barriers to provision of smoking cessation interventions in hospital inpatient settings. J Smok Cessat.

[52] Latimer A, Green K, Schmid K, Tomasone J, Abrams S, Cummings K (2010). The identification of framed messages in the New York State Smokers’ Quitline materials. Health Educ Res.

[53] McEwen A, West R, Preston A (2006). Triggering anti-smoking advice by GPs: mode of action of an intervention stimulating smoking cessation advice by GPs. Patient Educ Counsel..

