# Supplementary figures and images for: A personalized biomedical risk assessment infographic for people who smoke with COPD: a qualitative study
Source: Addict Sci Clin Pract. 2022 Jan 6;17:1. doi: 10.1186/s13722-021-00283-1 (PMC8734321; doi:10.1186/s13722-021-00283-1)

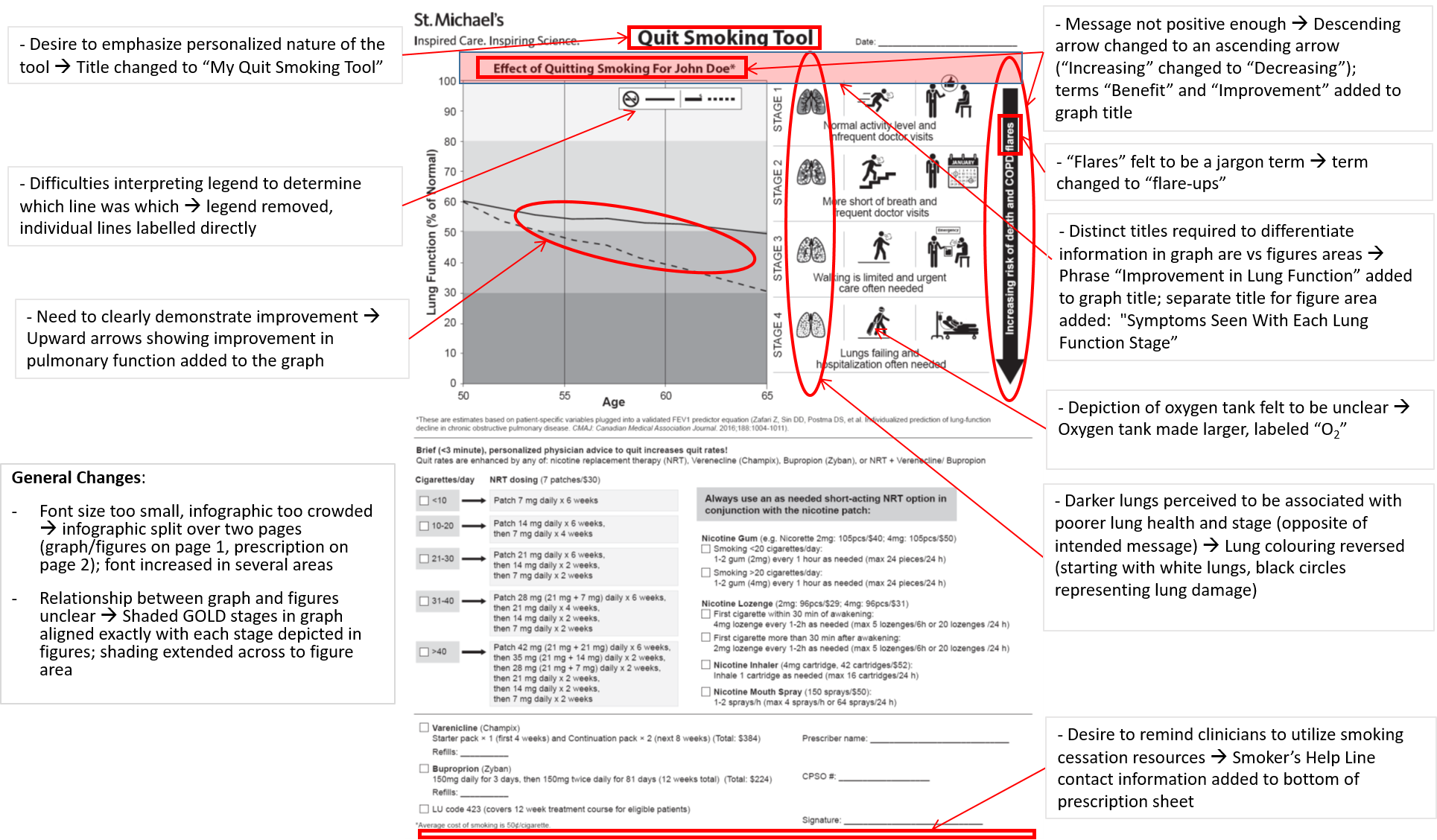

Supplement: Supplementary file 1 — Additional file 1: Serial changes to infographic in rapid-cycle design process. John Doe infographic is shown for simplicity; a similar Jane Doe infographic was also assessed. (A) 1-page infographic used in focus group 1, user comments and corresponding changes made. (B) 2-page infographic used in focus group 2, user comments and corresponding changes made. (C) 2-page infographic used in focus group 3, user comments and corresponding changes made. (D) 2-page infographic used in focus group 4, user comments and corresponding changes made. [file 13722_2021_283_MOESM1_ESM.zip › Supplementary Figure 1A.png]

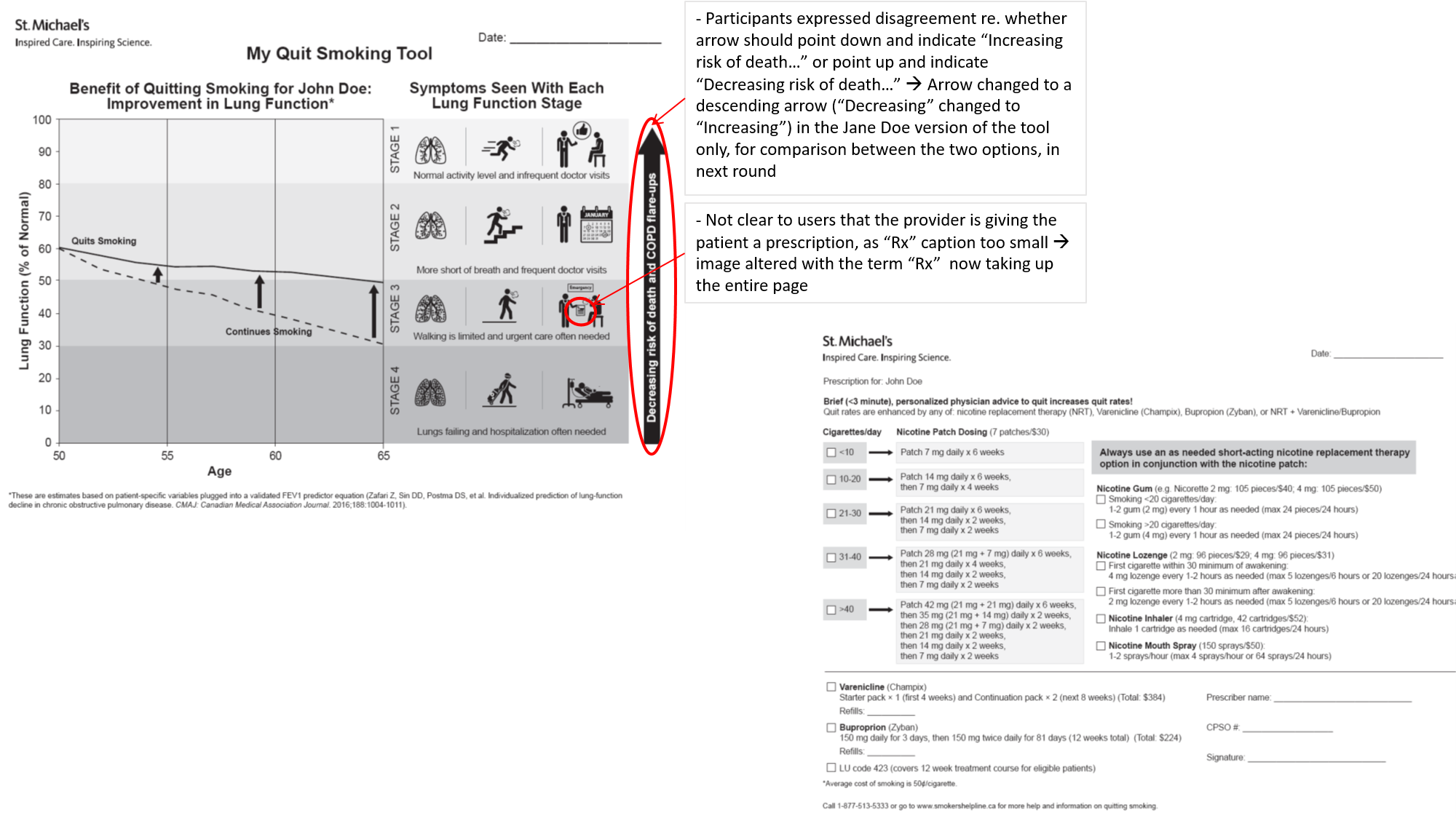

Supplement: Supplementary file 1 — Additional file 1: Serial changes to infographic in rapid-cycle design process. John Doe infographic is shown for simplicity; a similar Jane Doe infographic was also assessed. (A) 1-page infographic used in focus group 1, user comments and corresponding changes made. (B) 2-page infographic used in focus group 2, user comments and corresponding changes made. (C) 2-page infographic used in focus group 3, user comments and corresponding changes made. (D) 2-page infographic used in focus group 4, user comments and corresponding changes made. [file 13722_2021_283_MOESM1_ESM.zip › Supplementary Figure 1B.png]

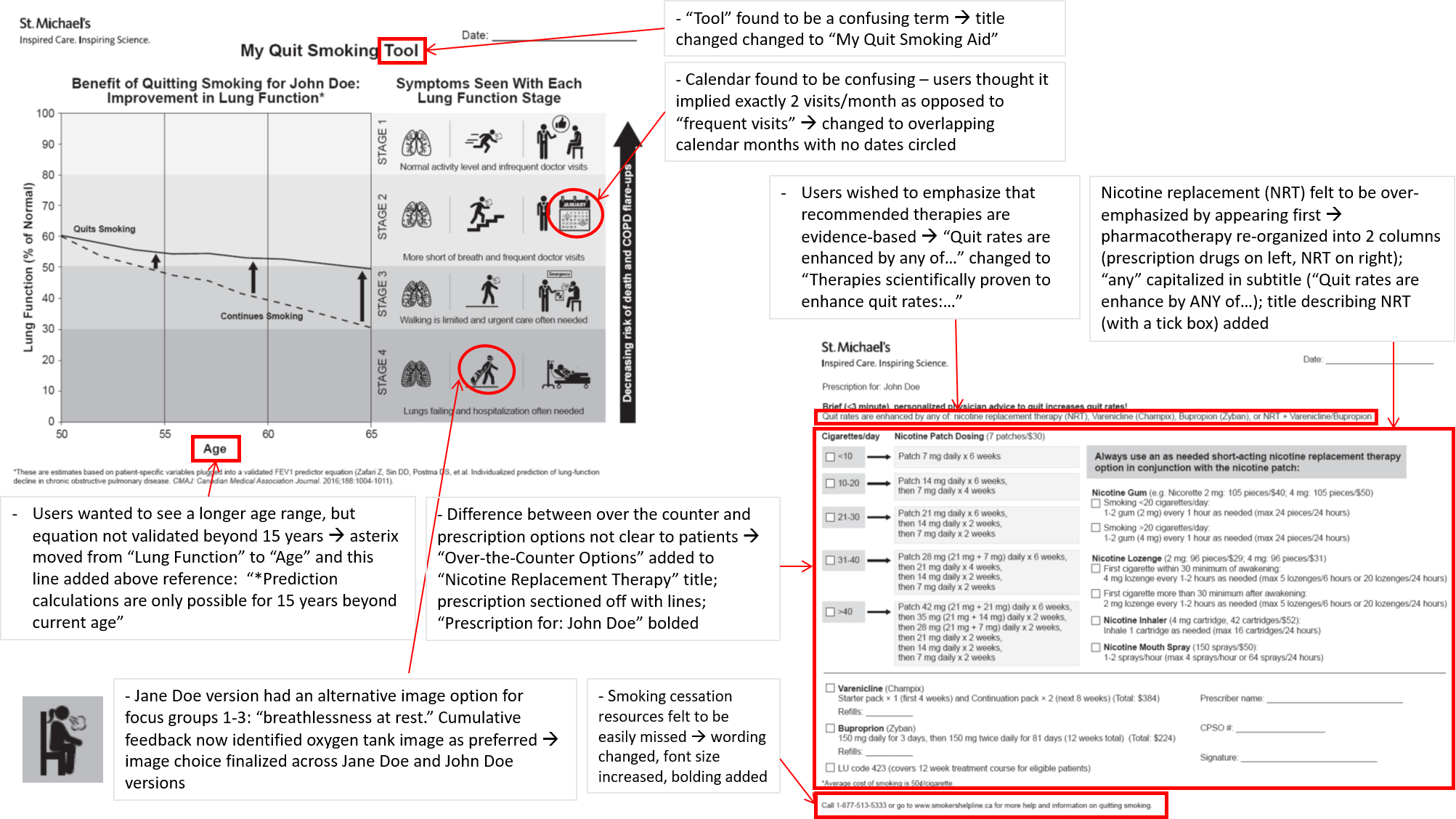

Supplement: Supplementary file 1 — Additional file 1: Serial changes to infographic in rapid-cycle design process. John Doe infographic is shown for simplicity; a similar Jane Doe infographic was also assessed. (A) 1-page infographic used in focus group 1, user comments and corresponding changes made. (B) 2-page infographic used in focus group 2, user comments and corresponding changes made. (C) 2-page infographic used in focus group 3, user comments and corresponding changes made. (D) 2-page infographic used in focus group 4, user comments and corresponding changes made. [file 13722_2021_283_MOESM1_ESM.zip › Supplementary Figure 1C.png]

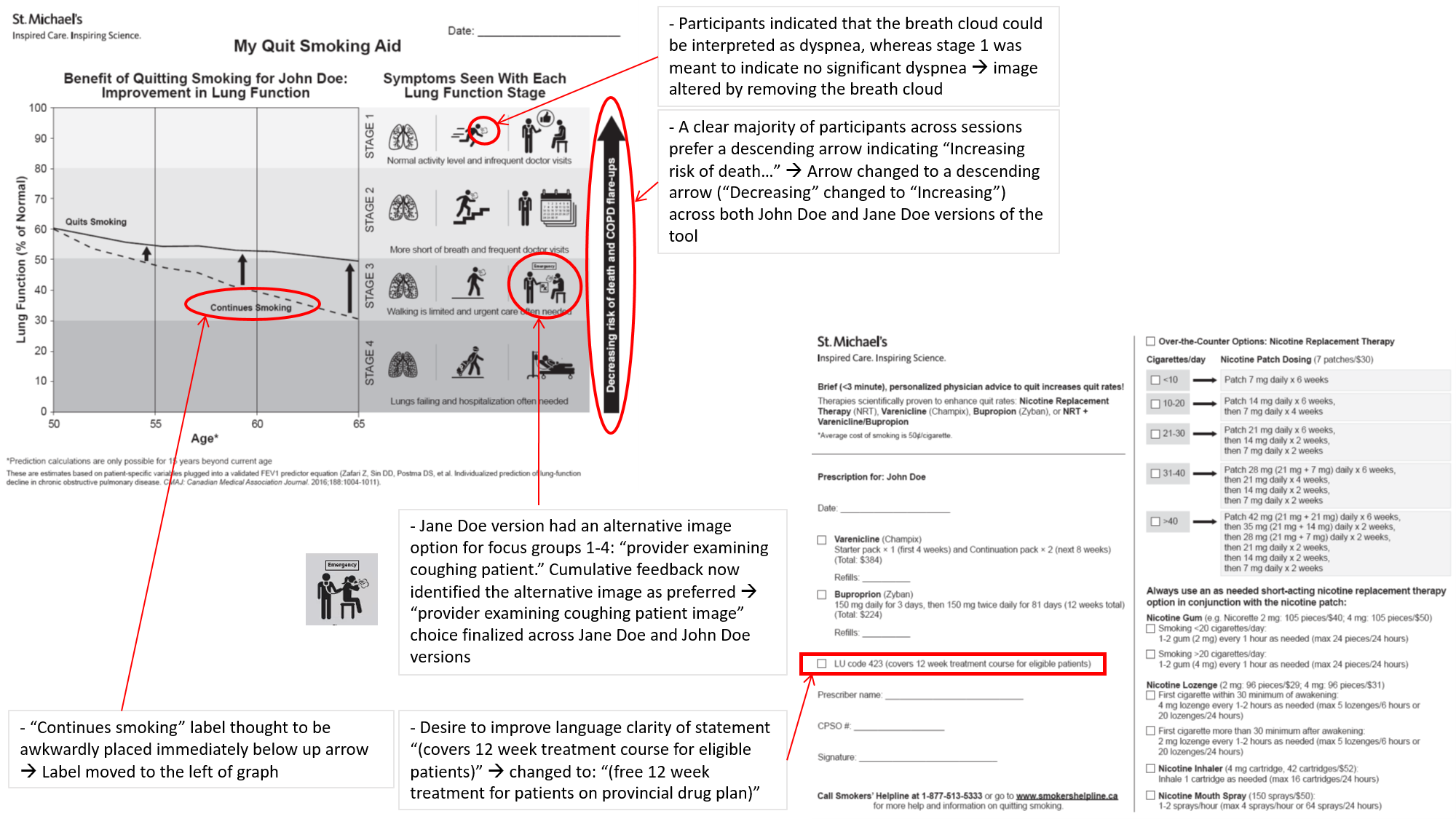

Supplement: Supplementary file 1 — Additional file 1: Serial changes to infographic in rapid-cycle design process. John Doe infographic is shown for simplicity; a similar Jane Doe infographic was also assessed. (A) 1-page infographic used in focus group 1, user comments and corresponding changes made. (B) 2-page infographic used in focus group 2, user comments and corresponding changes made. (C) 2-page infographic used in focus group 3, user comments and corresponding changes made. (D) 2-page infographic used in focus group 4, user comments and corresponding changes made. [file 13722_2021_283_MOESM1_ESM.zip › Supplementary Figure 1D.png]
